# Impact of educational video on performance in robotic simulation training (TAKUMI-1): a randomized controlled trial

**DOI:** 10.1007/s11701-023-01556-4

**Published:** 2023-03-11

**Authors:** Kosei Takagi, Nanako Hata, Jiro Kimura, Satoru Kikuchi, Kazuhiro Noma, Kazuya Yasui, Tomokazu Fuji, Ryuichi Yoshida, Yuzo Umeda, Takahito Yagi, Toshiyoshi Fujiwara

**Affiliations:** Department of Gastroenterological Surgery, Okayama University Graduate School of Medicine, Dentistry, and Pharmaceutical Sciences, 2-5-1 Shikata-cho, Kita-ku, Okayama, 700-8558 Japan

**Keywords:** Virtual reality, Robotic simulations, Educational video, Robotic surgery, Learning curve, Cumulative sum analysis

## Abstract

The use of virtual reality for simulations plays an important role in the initial training for robotic surgery. This randomized controlled trial aimed to investigate the impact of educational video on the performance of robotic simulation. Participants were randomized into the intervention (video) group that received an educational video and robotic simulation training or the control group that received only simulation training. The *da Vinci*^®^ Skills Simulator was used for the basic course, including nine drills. The primary endpoint was the overall score of nine drills in cycles 1–10. Secondary endpoints included overall, efficiency, and penalty scores in each cycle, as well as the learning curves evaluated by the cumulative sum (CUSUM) analysis. Between September 2021 and May 2022, 20 participants were assigned to the video (*n* = 10) and control (*n* = 10) groups. The video group had significantly higher overall scores than the control group (90.8 vs. 72.4, *P* < 0.001). Significantly higher overall scores and lower penalty scores were confirmed, mainly in cycles 1–5. CUSUM analysis revealed a shorter learning curve in the video group. The present study demonstrated that educational video training can be effective in improving the performance of robotic simulation training and shortening the learning curve.

**Supplementary Information:**

The online version contains supplementary material available at 10.1007/s11701-023-01556-4.

## Introduction

An increasing number of procedures are being performed as robotic surgery in the fields of gastroenterology, urology, and gynecology [1]. Robotic surgery can provide various advantages, including high-definition 3D views, a crystal-clear surgical field view, tremor-filtration technology, and articulated instruments. Specific surgical skills and training are required to be independent robotic surgeons. Hence, the structured training program for robotic surgery has been developed with the aim of translating skill from training to clinical practice in the operating room [2, 3]. In our training model that consisted of simulation, biotissue and video training, the use of virtual reality simulation played an important initial role in educating experienced surgeons and surgical trainees. The importance of virtual reality simulator training in robotic surgery has been advocated [4–7]. However, to date, only a few studies have investigated the effects of educational videos on the performance of robotic simulation training.

This study aimed to investigate the impact of educational video on performance of robotic simulation training, called the Training program in Okayama University for minimally invasive surgery (TAKUMI-1). Furthermore, the effect of the educational video on the learning curve for robotic simulation training was evaluated using a cumulative sum (CUSUM) analysis.

## Methods

### Trial design

We performed a randomized controlled trial with two parallel training groups; one group received an educational video and robotic simulation training (video group), while the other received simulation training alone (control group). The study protocol was approved by the institutional ethics committee of (No: 2108–017) and was registered at the University Hospital Medical Information Network (No: UMIN000045495). The Consolidated Standards of Reporting Trials (CONSORT) guidelines were followed [8]. Written informed consent was obtained from all participants prior to enrollment and randomization.

### Participants

Robotic surgery trainees in our department were recruited between September 2021 and May 2022. The participants included experienced surgeons and surgical trainees who had no experience with robotic simulation training.

### Robotic simulation training

This study was performed using a *da Vinci*^®^ Skills Simulator (Intuitive Surgical Inc., Sunnyvale, CA, USA). The *da Vinci*^®^ Skills Simulator for the basic course, including nine drills, was used for this trial: drill 1, sea spikes 1; drill 2, sea spikes 2; drill 3, camera targeting 1; drill 4, suture sponge 1; drill 5, thread the ring; drill 6, energy switching 1; drill 7, ring and rail 1; drill 8, needle targeting; and drill 9, 30° scope swap (Supplementary Table 1). The exercise goals for each drill are listed in Supplementary Table 1.

### Intervention

Educational videos on the tips and tricks of each drill were created by the principal investigator (KT) (Supplementary Table 1). The participants in both groups received robotic simulation training. However, the video group received an additional educational video training for a few hours prior to starting the robotic simulation. All participants had to perform the nine drills consecutively as one cycle, and complete 10 cycles in total.

### Primary and secondary endpoints

Assessment outcomes for each drill included overall, efficiency, and penalty scores. Efficiency scores were calculated by evaluating the time to completion, economy of motion, or master workspace range. Penalty scores consisted of excessive force use, instruments out of view, drops, instrument and cone collisions, incorrect rings, missed targets, and misapplied energy time. The overall scores were calculated by subtracting the penalty from the efficiency scores.

The primary endpoint was the overall score of all drills in cycles 1–10 in both groups. The secondary endpoint included the overall, efficiency, and penalty scores in each cycle and drill, as well as the learning curves between the groups.

### Sample size and randomization

The sample size was calculated based on the primary end point. Assuming a difference in the mean overall scores between the groups of 15 with a standard deviation of 10 according to our unpublished data, this study required a total sample size of 20 participants (10 participants for each group) to achieve a power of 90% and an alpha error of 5% (two-sided).

Randomization was performed by the principal investigator using a random number (Excel RAND) function. The participants were randomly divided into two groups: video and control.

### Statistical analysis

Statistical analysis and sample size calculation were performed using the JMP 11.2.0 software (SAS Institute, Cary, NC, USA). First, the overall, efficiency, and penalty scores of all drills in cycles 1–10 were compared between the video and control groups. Subsequently, the overall, efficiency, and penalty scores in each cycle as well as each drill were analyzed and compared between both groups. Moreover, the detailed penalty scores for each drill were compared between the groups. Finally, a CUSUM analysis was performed to compare the learning curves between the groups. The cumulative sums of the differences in each drill from the total “time to completion” were calculated based on the cycle. A pooled mean CUSUM was plotted to summarize the results [9, 10]. Data are presented as the mean (standard deviation) for continuous variables. Differences between groups were evaluated using Student’s *t* test or Mann–Whitney *U* test for continuous variables. Statistical significance was set at *P* < 0.05.

## Results

### Participant flow

**Fig. 1:**
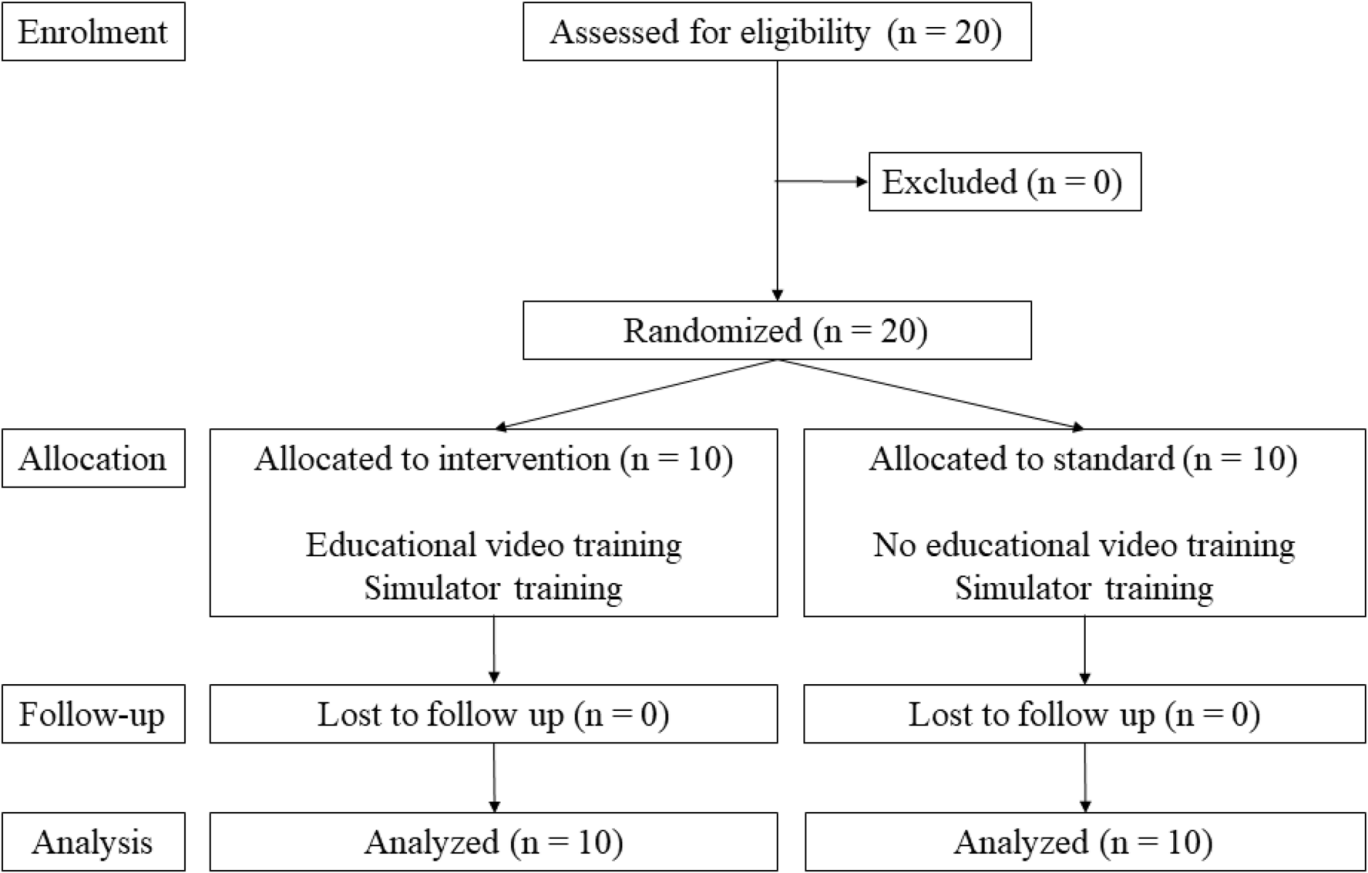
CONSORT flow diagram of the trial

The CONSORT flow diagram is shown in Fig. 1. A total of 20 participants were assigned either to the video (*n* = 10) or the control (*n* = 10) group (Table 1). Their specialities included gastrointestinal (*n* = 10) or hepatopancreatobiliary surgery (*n* = 10). All participants completed 100% of the curriculum in both groups.

**Table 1:** Baseline participant characteristics

	Total (*n* = 20)	Video group (*n* = 10)	Control group (*n* = 10)
Age (years)	34.5 (3.9)	33.8 (4.2)	35.1 (3.7)
*Gender*			
Male	18	9	9
Female	2	1	1
*Handedness*			
Right	19	9	10
Left	1	1	0
*Specialty*			
GI	10	6	4
HPB	10	4	6
Post-graduate year (years)	9.5 (3.9)	8.8 (4.2)	10.1 (3.7)

Data are presented as means (standard deviation)

*GI* gastrointestinal surgery, *HPB* hepatopancreatobiliary surgery

### Outcomes

The overall, efficiency, and penalty scores of all drills in cycles 1–10 between the video and control groups are shown in Table 2. The video group had significantly higher overall scores than the control group (90.8 vs. 72.4; *P* < 0.001).

**Table 2:** Drill scores in cycles 1–10 for the video and control groups

	Video group (*n* = 10)	Control group (*n* = 10)	*P* value
Overall score	90.8 (14.9)	72.4 (19.7)	< 0.001
Efficiency score	93.6 (12.3)	75.4 (16.8)	< 0.001
Penalty score	2.8 (5.9)	3.0 (8.27)	< 0.001

Data are presented as means (standard deviation)

*The overall scores* (0–100) were calculated by subtracting the penalty scores from the efficiency scores. *Efficiency scores* were calculated by evaluating the time to completion, economy of motion, or master workspace range. *Penalty scores* consisted of excessive force, instruments out of view, drops, instrument collisions, incorrect rings, cone collisions, missed targets, and misapplied energy time

**Fig. 2:**
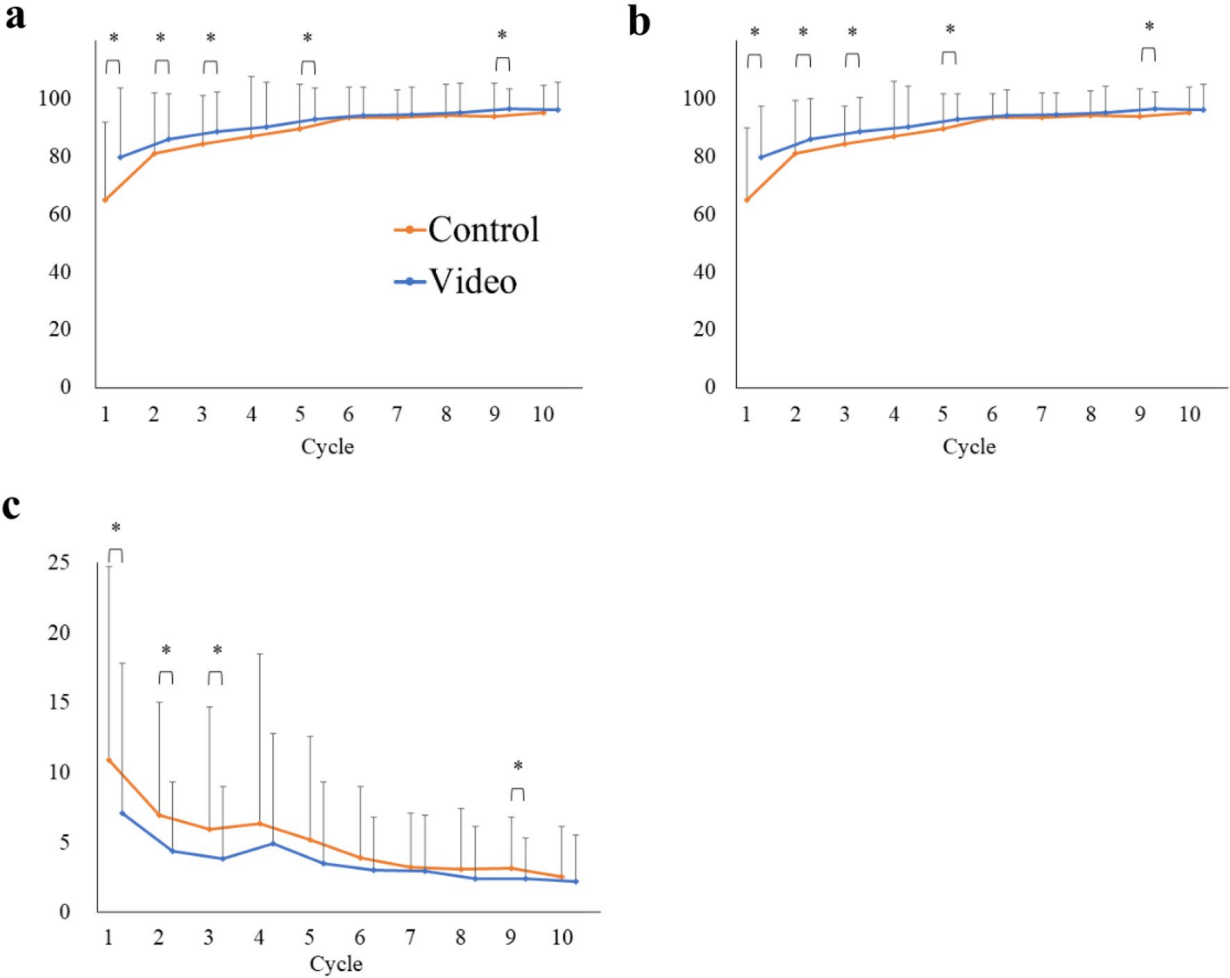
**a** Overall, **b** efficiency, and **c** penalty scores in each cycle for the video and control groups. *P < 0.05

**Fig. 3:**
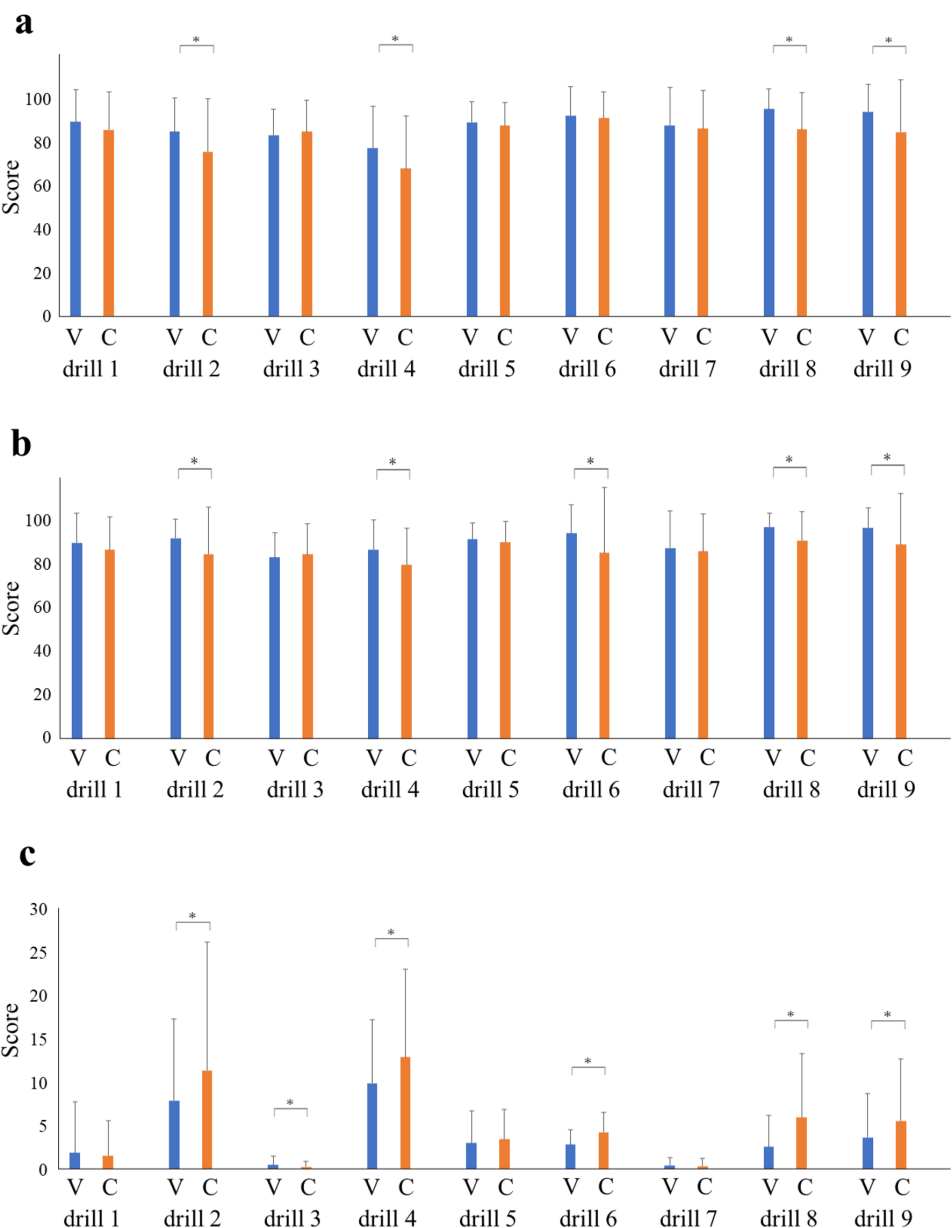
**a** Overall, **b** efficiency, and **c** penalty scores in each drill for the video and control groups. *V* video group, *C* control group. *P < 0.05

The overall, efficiency and penalty scores in each cycle between the groups are shown in Fig. 2. The trend of significantly higher overall and lower penalty scores was confirmed mainly in cycles 1–5. The overall, efficiency and penalty scores for each drill between the groups are shown in Fig. 3. The overall score was significantly higher in the video group than in the control group in four out of the nine drills (drills 2, 4, 8, and 9). The video group had significantly lower penalty scores than the control in six drills (drills 2, 3, 4, 6, 8, and 9). The video group had lower penalty scores in collisions and out-of-view instruments than in the control group (Supplementary Fig. 1).

**Fig. 4:**
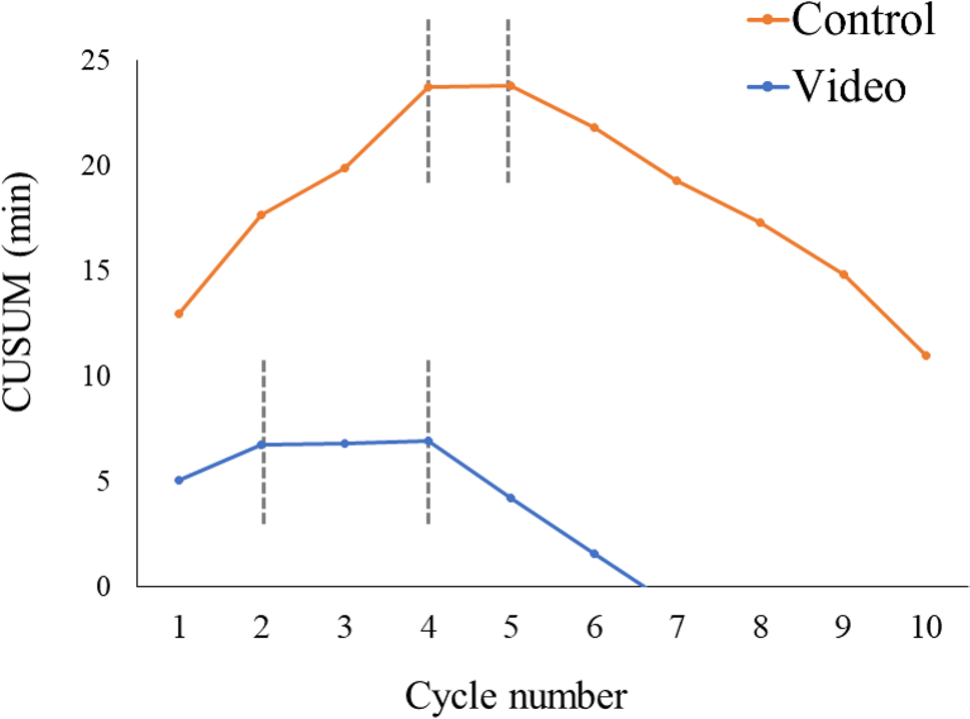
The cumulative sum (CUSUM) analysis of the total “time to completion” in each cycle

The CUSUM analysis of the total “time to completion” in each drill between the groups is depicted in Fig. 4. The video group reached a plateau within two cycles, and improved after four cycles. In contrast, the control group required 4 cycles to reach a plateau.

## Discussion

This is the first randomized controlled trial to investigate the impact of educational videos on the performance of robotic simulation training. We found that educational video training improved the performance in robotic simulation. Moreover, CUSUM analysis confirmed the positive effect of the educational video on shortening the learning curve.

Educational video should be exemplary for early training in robotic surgery and have the potential to maximize trainees’ learning and skill improvement [11]. Video training could allow preparation prior to starting surgical training. Furthermore, a video-based education system can provide best practices by improving surgical quality and reducing learning curves for complex robotic procedures [12]. Accordingly, video-based education should be an effective method in surgical education [13, 14].

Regarding the evidence on robotic simulation training, several randomized controlled trials have been performed to investigate the effect of an affordable surgical robot simulator or procedural virtual reality, showing effective impact in improving robotic surgical skills [15, 16]. However, few studies have examined the effects of educational videos on the performance of robotic simulation training so far. Therefore, our study observed novel findings that demonstrated the efficiency of educational video for improving the performance in robotic simulation.

We determined that the overall scores of all drills, would be the best index for estimating the impact of educational video training. The mean overall scores in the video group were 18.4 times higher than those in the control group, probably due to educational video training.

Participants in the control group improved their overall, efficiency and penalty scores through self-learning, and eventually reached the same level as that of the video group. However, the video group had significantly higher efficiency and lower penalty scores, especially during the initial phase. We propose that participants in the video group had improved their scores through both educational video training and self-learning. The penalty scores in the video group were lower throughout the drills, especially in collisions and out-of-view instruments (Fig. 3 and Supplementary Fig. 1), than in the control group. This may be explained by the fact that educational video training can increase the ability to recognize spatial cognition.

CUSUM analysis has been used to evaluate the learning curves for surgical procedures [9, 10]. As “time to completion” is a useful predictor of efficiency scores, we believe that the total time required to complete each drill is a good indicator of the evaluation of the learning curves. Interestingly, our learning model using CUSUM analysis showed different learning curves between the two groups. The findings suggested a significant effect of educational video training on shortening learning curves.

Considering the positive effect of video training, our findings indicate that surgical video training could lead to improved robotic surgery performance in the clinical setting. Therefore, a structured training model for robotic surgery, that includes simulation, biotissue, and video training, should improve surgical skills and shorten learning curves [17, 18]. A recent meta-analysis demonstrated that surgical skills acquired through robotic simulator training can be translated to the operating room with regards to time and technical performance [4]. Moreover, it suggested potential benefits that could justify simulator training in structured robotic training programs and emphasized the role of simulation training before performance in a real operating room.

The present study had several limitations. Although this was a randomized controlled trial, the sample size was small. Further studies with larger sample sizes are required to externally validate our findings. The association between the performance of simulation training and performance in a real operating room has not yet been investigated. Therefore, future studies focusing on these are required to confirm the effect of simulation training on performance in the clinical setting. Finally, the effect of educational video on each drill was unclear. A further investigation should be helpful to understand which surgical skills can be improved through the video training.

In conclusion, this study demonstrated that educational video training can be effective in improving the performance of robotic simulation training and shortening the learning curve.

## Supplementary Information

Below is the link to the electronic supplementary material.

Supplementary file1 (DOCX 6196 KB)
