# Supplementary material for: Impact of educational video on performance in robotic simulation training (TAKUMI-1): a randomized controlled trial
Source: J Robot Surg. 2023 Mar 11;17(4):1547–53. doi: 10.1007/s11701-023-01556-4 (PMC10374749; doi:10.1007/s11701-023-01556-4)

**Supplementary Table 1.** Exercise goals and educational video for each drill in the *da Vinci*^®^ Skills Simulator for basic course (Intuitive Surgical Inc., Sunnyvale, CA).

| Drill |  | Exercise goals | URL |
| --- | --- | --- | --- |
| 1 | Sea spikes 1  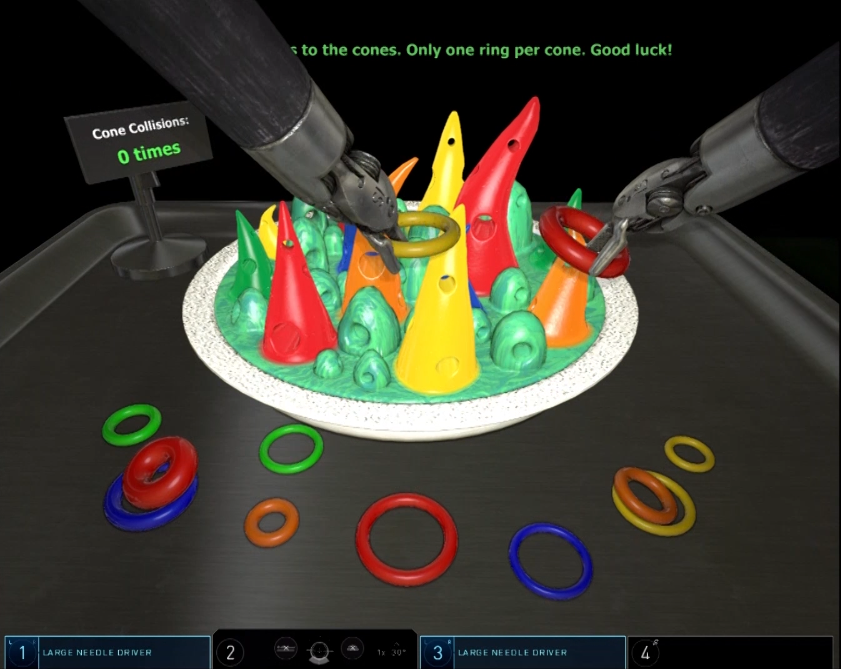 | Place the colored rings onto the matching colored cones. As there are multiple rings and multiple cones with the same color, place only one ring on each cone. | https://youtu.be/zVD20TYKsOk |
| 2 | Sea spikes 2  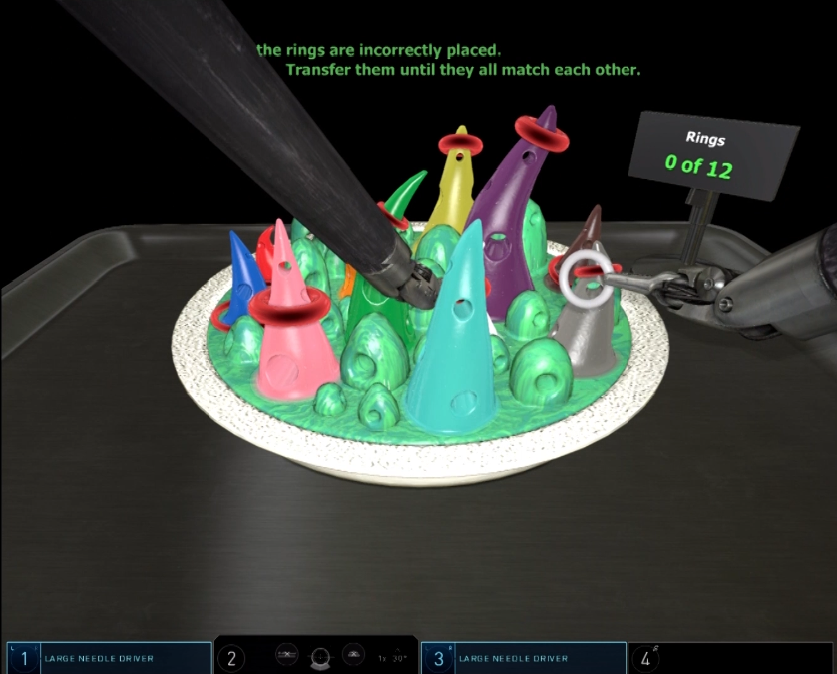 | The 12 uniquely-colored rings are incorrectly matched to the 12 uniquely-colored cones. Pick up and place each ring with its matching colored cone by using both arms to facilitate the transfer. | https://youtu.be/8R5niiFsj7Q |
| 3 | Camera targeting 1  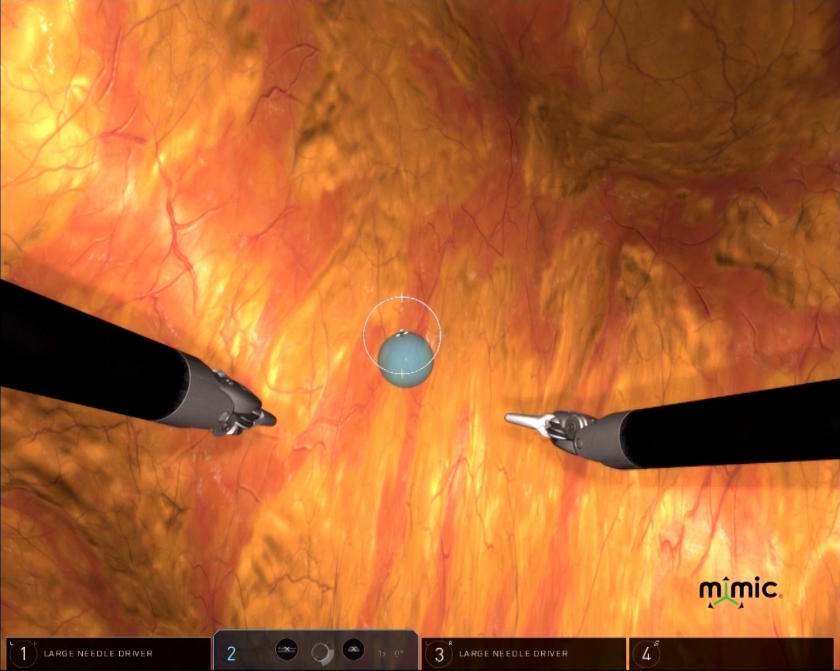 | Move the camera to follow the onscreen guide until you position each light blue sphere in the center of your screen’s target. | https://youtu.be/_cWPEpBabuA |
| 4 | Suture sponge 1  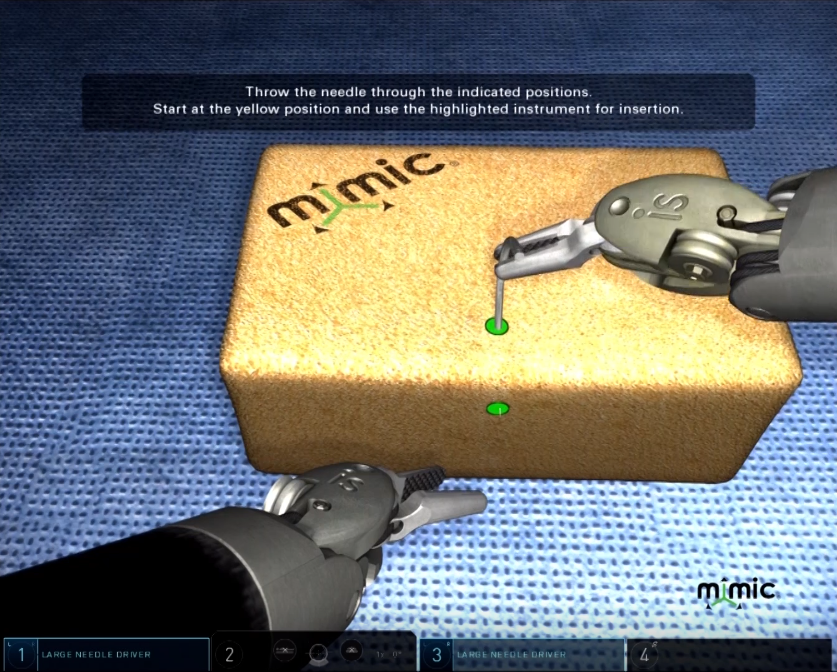 | Learn to control a needle when passing it between instruments. Insert and extract the needle through several pairs of targets on the edge of a sponge. | https://youtu.be/Mo43SYdT1H0 |
| 5 | Thread the ring  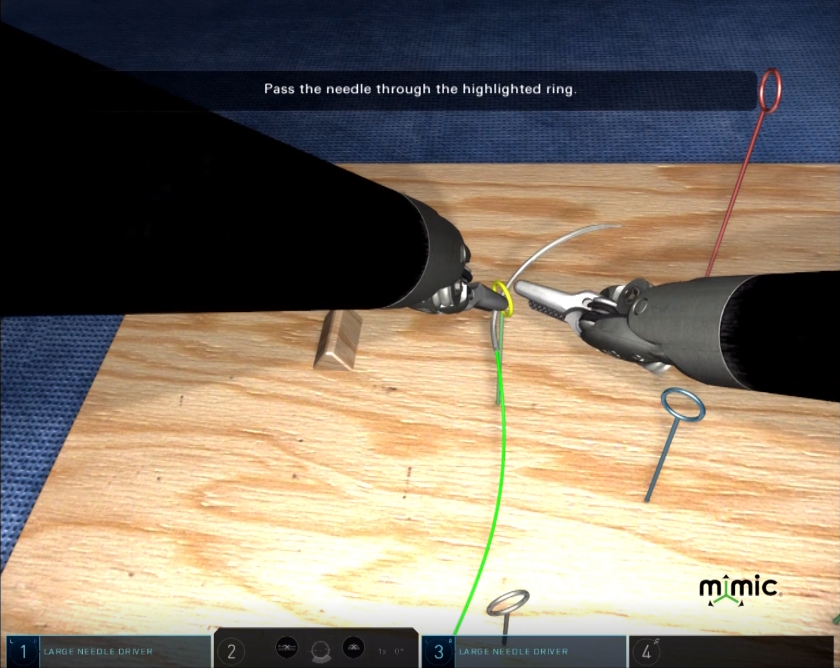 | Pick up the needle and suture and pass them through each subsequent highlighted ring. | https://youtu.be/C5JMh2d6m1E |
| 6 | Energy switching 1  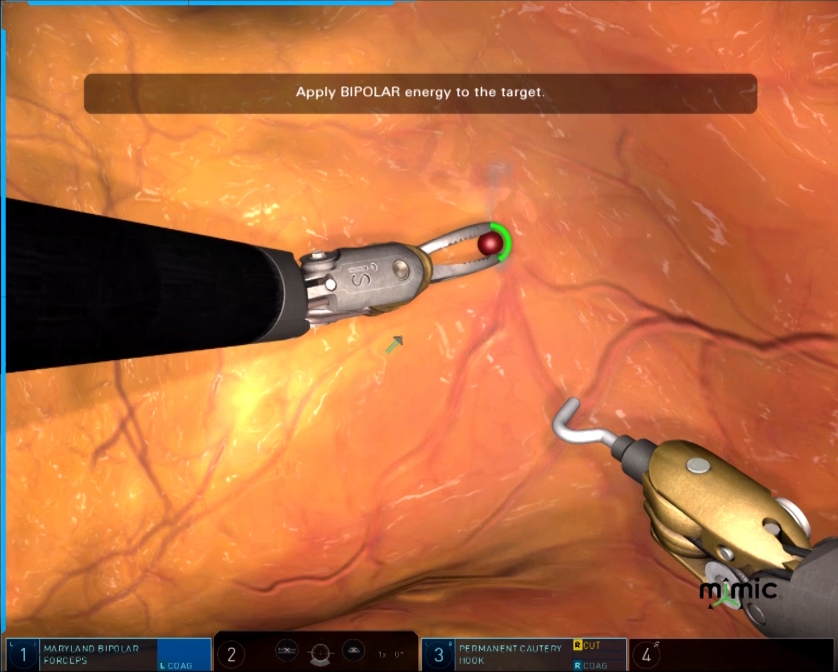 | Make contact with each on-screen targets using the appropriate instrument and apply the correct energy source as directed. | https://youtu.be/_ZVZm9aNS6k |
| 7 | Ring and rail 1  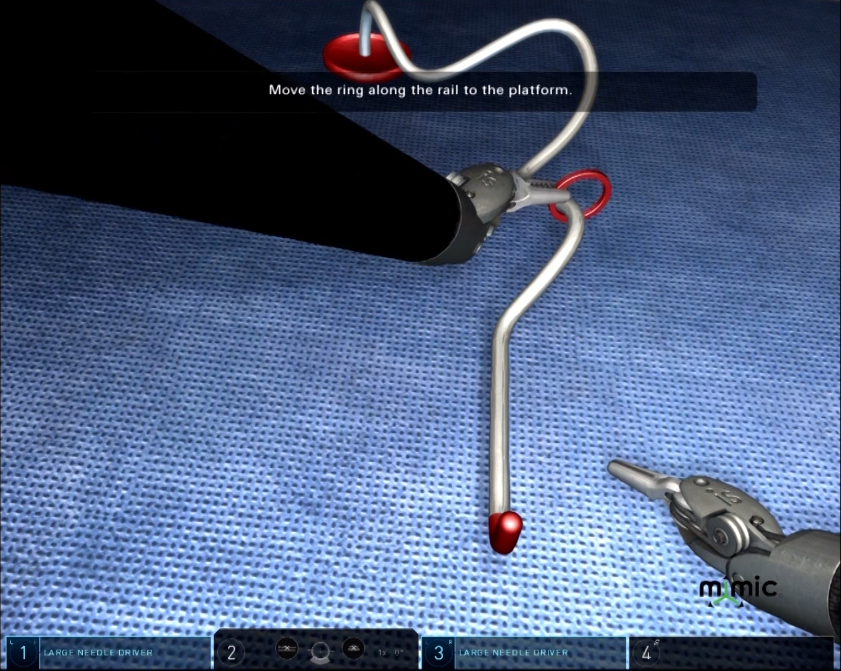 | Pick up the ring off the floor and guide it along the curving rail. | https://youtu.be/Pwz3yrDqqbA |
| 8 | Needle targeting  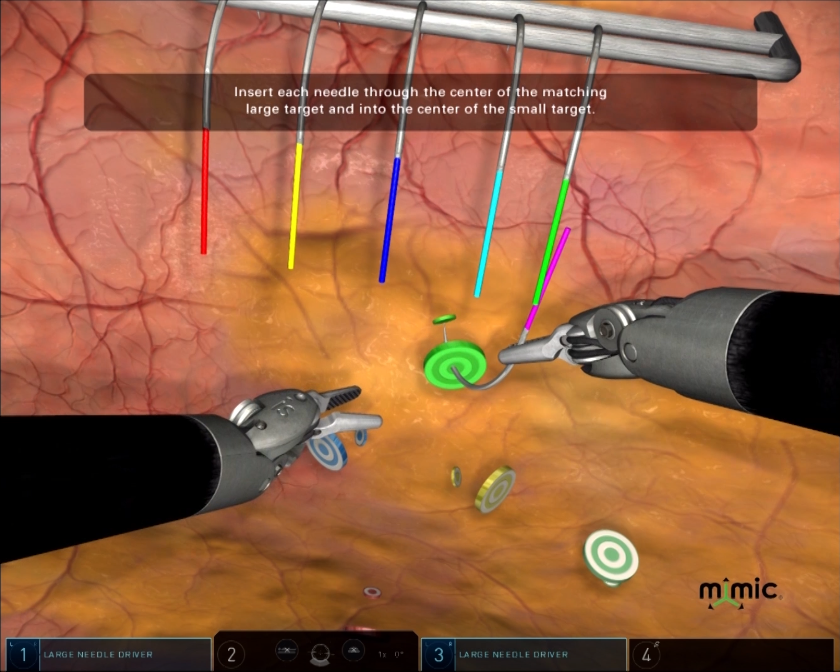 | Remove each needle from the rack and accurately insert it through the pair of matching colored rings. | https://youtu.be/hr500X8P0zk |
| 9 | 30 degree scope swap  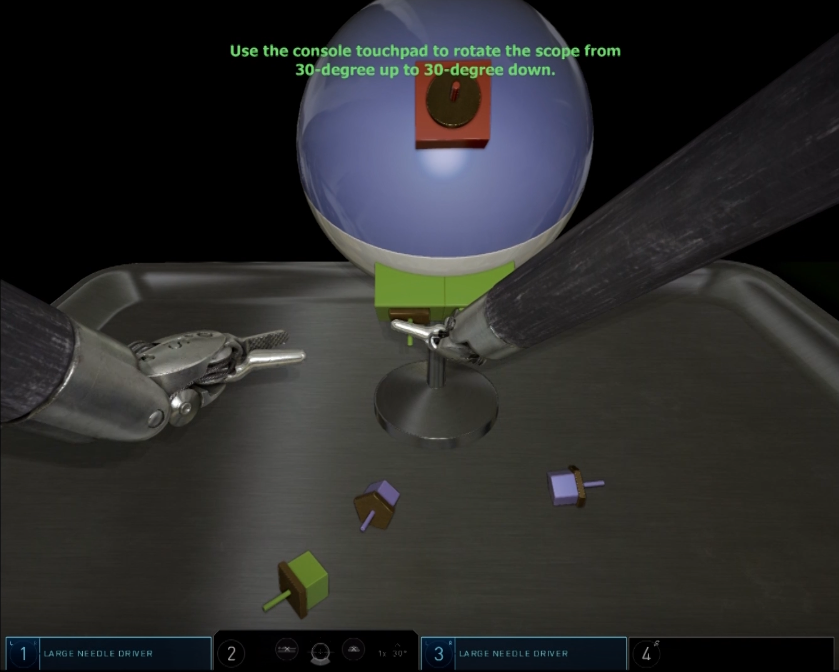 | Pick each shape up from the tray and put it into its corresponding compartment on the exercise sphere. | https://youtu.be/6xMum7A0fK4 |

**Supplementary Figure 1.** Detailed penalty scores in each drill for the video and control groups. (a) drill 1, (b) drill 2, (c) drill 3, (d) drill 4, (e) drill 5, (f) drill 6, (g) drill 7, (h) drill 8, (i) drill 9. *P < 0.05


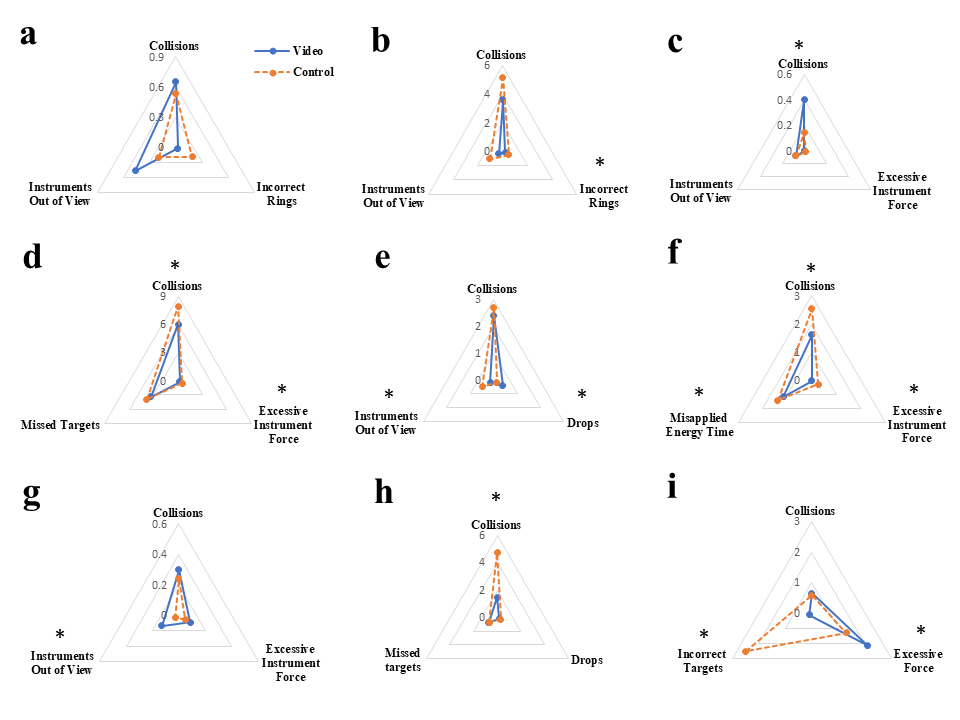

Supplement: Supplementary file 1 — Supplementary file1 (DOCX 6196 KB) [file 11701_2023_1556_MOESM1_ESM.docx]
